# Cochineal carmine adsorbed on layered zinc hydroxide salt applied on mortadella to improve color stability

**DOI:** 10.1016/j.crfs.2021.10.006

**Published:** 2021-10-22

**Authors:** Gabriela Cavalca Ongaratto, Gabriela Oro, Daneysa Lahis Kalschne, Ana Cristina Trindade Cursino, Cristiane Canan

**Affiliations:** a Departamento de Alimentos, Universidade Tecnológica Federal Do Paraná, Medianeira, Paraná, Brazil; b Departamento de Química, Universidade Tecnológica Federal Do Paraná, Medianeira, Paraná, Brazil

**Keywords:** Curing salt, Layered zinc hydroxide salt, Natural dye, Stability color, Meat product

## Abstract

The pink/reddish color meat products characteristic of cured meat without the curing salts is a meat industry demand to serve consumers who are looking for healthy foods with the usual sensory characteristics. This study aimed to obtain and characterize a hybrid dye and use it as a replacer for curing salt in the production of pink/red color in mortadella stored for 40 days. A layered zinc hydroxide salt (ZHN) was obtained by alkaline precipitation to immobilize and increase cochineal carmine stability, obtaining the hybrid dye (ZHN-carmine) by ion exchange in aqueous solution. The ZHN-carmine was subjected to ultrasound to increase color intensity and reduce the amount of application. ZHN, cochineal carmine and ZHN-carmine were characterized by X-Ray Diffraction, Fourier-Transform Infrared Spectroscopy, Thermogravimetric Analysis and Differential Scanning Calorimetry. The ZHN-carmine was used in the mortadella elaboration traditionally prepared with nitrite/nitrate and/or carmine. In the characterization it was observed that carmine dye was adsorbed on the lamellar compound surface and over the mortadella storage, it ensures a more stable pink/reddish color than the others product formulations. A more intense color with lower L* and higher a* values was observed for mortadella added of ZHN-carmine ultrasound-assisted. Therefore, the lamellar matrix adsorbed with cochineal carmine may a suitable and useful alternative to obtain the pink/reddish color characteristic of cooked meat products by applying a natural hybrid dye.

## Highlights

Hybrid dye was obtained by adsorbing carmine dye on a layered zinc hydroxide salt
The hybrid-dye mortadella had a stable pink/reddish color characteristic
The ultrasound improved the color supplied by hybrid dye in mortadella
Hybrid dye may be used as curing salt replacer on mortadella without color loss

## Graphical abstract

**Figure:**
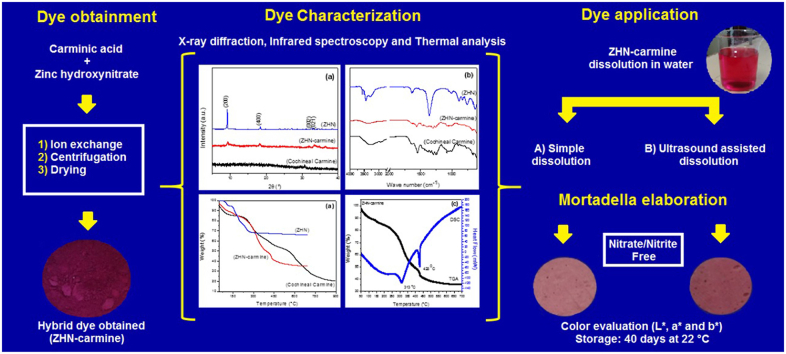

## 1 Introduction

Color is the first sensory characteristic observed by the consumer when choosing a food (Da Silva-Buzanello et al., 2017; Wu and Sun, 2013; Arvanitoyannis and Stratakos, 2012). It has a very powerful reference on quality, especially for meat and meat products. The pigmented compounds found in fresh meat, responsible for the red color are myoglobin, hemoglobin, muscle tissue proteins, and blood. Myoglobin may be oxidized by oxygen, with heme iron converted to the ferric state, forming dark brown metmyoglobin. Such a reversible reaction only occurs in fresh meat (Mancini and Hunt, 2005) and the addition of dyes is not permitted to avoid fraud and confusion to the consumer. The meat curing processing involves using either nitrite and/or nitrate salts to improve meat products' preservation due to the pronounced inhibitory effects of nitrite on anaerobic bacteria, namely *Clostridium botulinum*. Aside from acting as an antibotulinal agent, nitrite is also a strong oxidizer and is quickly transformed into nitric oxide, which reacts with the Fe^3+^ and Fe^2+^ of both myoglobin and metmyoglobin, respectively (Jo et al., 2020). It forms heat stable red-colored complexes, developing the appealing cherry-red color of cured meat, which consumers are used to (Viuda-Martos et al., 2009). However, there has been a growing demand for healthy foods with fewer additives, especially curing salts that may lead to carcinogens formation (Pöhnl, 2016). No replacement has yet been available on the market that may be capable to present both antimicrobial and color-forming effects in either raw or cooked meat products (Bedale et al., 2016). Studies have been carried out to inhibit *Clostridium* genus bacteria in meat products without using any nitrates and nitrites (Canan et al., 2018; Ferysiuk and Wójciak, 2020a; Sindelar et al., 2007). However, it is not possible to completely eliminate nitrite from the production process of cooked meat products without deteriorating the color (Ferysiuk and Wójciak, 2020b).

Color stability is also important during storage of meat products, which may be vacuum-packaged or modified atmosphere packing, and even exposed to light. The use of these two types of package are indispensable in meat processing, because it acts as an efficient tool on spoilage microorganism inhibition and to reduce the residual nitrite of processed meat. However, color stability during storage is a relevant factor that needs to be improved, regardless the processing technology used (Arvanitoyannis and Stratakos, 2012). Even using emerging technologies such as ultra-high pressure (UHP) (Orlien, 2017), ultrasound (Fallavena et al., 2020), and high-pressure carbon dioxide (HPCD) (Yan et al., 2016) a significantly color changes may occur after processing and/or storage.

The food industry, as well as the food dyes industrial production, have become quite relevant over the past decades. Tons of artificial and natural dyes are used on a daily basis, mainly to improve or change the products’ natural color (Agócs and Deli, 2011). One of the natural dye mostly used in meat products is carminic acid, permitted in several countries and by international legislations (Pöhnl, 2016). It is extracted from female *Dactylopius coccus* dry bodies, a cochineal insect species (Borges et al., 2012). Such dye is widely used in meat products embedded together with curing salts, to obtain the desired pink color. Despite the high stability of cochineal carmine regarding the cooking temperatures (Müller-Maatsch and Gras 2016), previous studies have indicated its instability under light (Bowers and Sobeck 2016), and acid pH (Ghidouche et al., 2013). Several techniques such as non-toxic hybrid pigments (Lima et al., 2009), and microencapsulation (Cai et al., 2019) have been used for greater natural dye stability. On the other hand, all efforts to find a safe and equivalent replacement for curing salts in terms of color have not yet been successful. Layered zinc hydroxide salts, such as hydrozincite and zinc or copper hydroxynitrate, are anionic exchangers, of which a part of the anions that interact by electrostatic forces may be exchanged for other anions of interest. For layered zinc hydroxide salt, the nitrate may be exchanged for cochineal carmine dye. Layered hydroxide salts have a low toxicity level, low production cost and are easily obtained and manipulated (Forano, 2004). There are numerous functions such as ultraviolet absorbers (Cursino et al., 2010) and alternative loads for polymeric composite materials (Da Silva et al., 2013). They are also used for vitamin intercalation and aroma adsolubilization, acting as food supplements conferring aroma, increasing the stability and nutritional properties of the product (Cursino et al., 2018) as well as providing chemical and biological protection for food-grade nisin aiming at antimicrobial use (Velázquez-Carriles et al., 2020). The carminic acid was intercalated into others layered structures, such as layered double hydroxide (LDH) or hydrotalcite and montmorillonite (Fournier et al., 2016; Kohno et al., 2009). Hybrid materials based on one organic dye, the carminic acid, and montmorillonite was obtained by Fournier et al. (2016). The carminic acid has also been intercalated in the hydrotalcite layer and the studies details its characterization and its increased in the photostability (Kohno et al., 2009).

The microbiological safety and color stability of meat products not added with any curing salts may only occur by adding another additive. For this, research to enable such practice is essential. It is assumed that stable natural dyes must be developed to achieve the desired color of meat products without adding any curing salts. In this study, the inorganic matrices such as layered zinc hydroxide salt adsorbed with natural cochineal carmine dye (ZHN-carmine) were obtained, characterized and applied in a nitrite-free mortadella production to increase its color stability on storage when compared to the color of traditionally produced mortadella (with nitrite/nitrate and/or carmine).

## 2 Materials and methods

### 2.1 Zinc hydroxynitrate (Zn_5_(OH)_8_(NO_3_)_2_.2H_2_O) obtainment

To obtain the hybrid dye (ZHN-carmine), layered zinc hydroxide salt (ZHN) was obtained by alkaline precipitation, as described by Forano (2004) and Wypych et al. (2005). 100 mmol L^-1^ zinc nitrate solution was prepared (Dinâmica, Indaiatuba, São Paulo, Brazil), and added with 1 mol L^-1^ sodium hydroxide (Dinâmica, Indaiatuba, São Paulo, Brazil) under vigorous stirring (Corning, 6796-420D, New York, USA), until reaching pH 6.8 (Even, PHS-3E). After 24 h stirring, the solid was separated by centrifugation (Cientec, CT 5000R, Belo Horizonte, Minas Gerais, Brazil) at 5000 rpm for 5 min at 25 °C, washed with distilled water and centrifuged. This washing process was repeated three times. The obtained solid (ZHN) was oven-dried (New Lab, NL 80, Piracicaba, São Paulo, Brazil) at 40 °C, macerated and stored for ion exchange reaction with cochineal carmine.

### 2.2 Ion exchange reaction to obtain hybrid dye (ZHN-carmine)

The ion exchange reaction was carried out as described by Forano (2004) and Wypych et al. (2005), with some modifications. 2.4 mmol of layered zinc hydroxide salt were added to a 4 mmol cochineal carmine solution (Carmine WS 52% Globenatural, Chorrillos, Peru), with final pH corrected to 7.0, with 2 mol L^-1^ hydrochloric acid (Dinâmica, Indaiatuba, São Paulo, Brazil) The dispersion remained under magnetic stirring (Corning, 6796-420D, New York, USA) for 7 days at 70 °C and was subsequently rinsed with distilled water and centrifuged at 5000 rpm (Cientec, CT 5000R, Belo Horizonte, Minas Gerais, Brazil) for 5 min at 25 °C. The process was repeated until obtain a light-pink residual water. After that, the obtained solid was dried in a vacuum desiccator with silica, then macerated and stored. In this way, a hybrid dye product named ZHN-carmine was obtained.

### 2.3 Samples characterization

The zinc hydroxynitrate (ZHN), cochineal carmine, and hybrid dye (ZHN-carmine) obtained were characterized by X-Ray Diffraction (XRD), using a PANalytical diffractometer, (Empyrean model, Westborough, USA) with CuKα = 1.5418 Å radiation source, 30 mA current and 40 kV voltage in the Interdisciplinary Laboratory of Physical Sciences at the Federal University for Latin American Integration, Foz do Iguaçu, Brazil. Fourier-Transform Infrared Spectroscopy (FTIR) analyses were carried out using a PerkinElmer spectrometer (Spectrum 100s model, Beaconsfield, UK) with an attenuated total reflectance (ATR) accessory with a zinc selenide crystal (ZnSe), and 4 scans accumulation ranging from 600 to 4000 cm^−1^ and 4 cm^−1^ resolution.

Thermogravimetric (TGA) and differential scanning calorimetry (DSC) were carried out using a PerkinElmer Thermal-Analyzer (STA 6000 model, Beaconsfield, UK) with a 20 mL min^-1^ oxygen flow rate and a 10 °C min^-1^ heating rate of up to 900 °C. The samples mass used ranged from 6 to 8 mg.

### 2.4 Hybrid dye (ZHN-carmine) applied on mortadella

Hybrid dye (ZHN-carmine) and cochineal carmine were applied and compared on mortadella color stability. The mortadella was produced with pork, beef and other additives and ingredients (Table 1) following the Brazilian Identity and Quality Standards (Brasil, 2000).

**Table 1:** Formulations for the preparation of mortadella added with hybrid dye (ZHN-carmine) or with cochineal carmine.

Raw material and ingredients	M1 (%)	M2 (%)	M3 (%)	M4 (%)
Pork and beef	72.00	72.00	72.00	72.00
Ice	20.00	20.00	20.00	20.00
Cassava starch C-400 (Cassava S/A)	3.00	3.00	3.00	3.00
Isolated soy protein (Proteíco®)	2.00	2.00	2.00	2.00
Sodium tripolyphosphate (Ibrac®)	0.25	0.25	0.25	0.25
Monosodium glutamate (Ajinomoto®)	0.10	0.10	0.10	0.10
Sodium erythorbate (Ibrac®)	0.25	0.25	0.25	0.25
Condiments (Conditec®)	0.52	0.52	0.52	0.52
Salt (Diana®)	1.630	1.625	1.855	1.855
Nitrate/Nitrite (Ibrac®)	0.25	0.25	–	–
Cochineal carmine (Globenatural®)	–	0.005	–	–
Hybrid dye (ZHN-carmine)	–	–	0.025	0.025
Total	100.00	100.00	100.00	100.00

M1, mortadella with curing salts and without dyes; M2, mortadella with curing salts and cochineal carmine; M3, mortadella without curing salts and with hybrid dye (ZHN-carmine); M4, mortadella without curing salts and with hybrid dye (ZHN-carmine) dissolved by ultrasound.

The hybrid dye (ZHN-carmine) content added to M3 and M4 mortadella formulations were higher in percentage when taking into account the total compound mass, however, the cochineal carmine amount was equivalent to M2 mortadella formulation. The mortadella preparation was performed using a cutter (Mado, Garant MTK 661 model, Dornhan, Germany). The hybrid dye (ZHN-carmine) was submitted to an ultrasound treatment using a Vibra Cell ultrasonic processor (Sonics, VC-505, Newtown, USA), at a 37 kHz frequency, 100% amplitude for 4 min to disaggregate and better disperse the adsorbed pigment (Silva et al., 2013). Cochineal carmine was exempted from such procedure due to its highwater solubility. All mortadella formulations were processed in a pilot plant according to industrial procedures. The frozen meat (−1 °C) was placed into a cutter (Mado, MTK-661, Dornhan, Germany) with NaCl and comminuted for 2–3 min at low speed to extract myofibrillar proteins. The other additives were added slowly, and the temperature of the meat batters never exceeded 7 ± 1 °C. Meat emulsion (300 g) was stuffed into nype-type artificial casings comprised of polyamide and polyethylene (PA/PE) with a 70 mm diameter and water-heated at 80 °C until reaching 73 °C internal temperature. A thermocouple probe (Asko, AK16S, São Leopoldo, Rio Grande do Sul) placed in the geometric center of the mortadella was used to monitor the product's temperature. After cooking, the mortadella was cooled in running water for 20 min and stored for 40 days at room temperature (22 ± 1 °C).

### 2.5 Mortadella color stability evaluation during storage

Samples from each mortadella formulation were randomly selected for each analysis day (0, 8, 16, 24, 32, and 40 days), and the slices’ surface color was determined at three different spots with the CIELAB system using a colorimeter (CR 400, Konica Minolta, Osaka, Japan) calibrated with a D65 standard illuminant and a 10° angle. L* (lightness; 100 = white, 0 = black), a* (redness; +, red; −, green), and b* (yellowness; +, yellow; −, blue) values were obtained. The results were presented as average ± average standard deviation, submitted to analysis of variance (one-way, ANOVA) followed by the Tukey test (p ≤ 0.05) using the Statistica 7.0 software.

### 2.6 Statistical analysis

The entire experiment was repeated three times (n = 3). A randomized block design was used to evaluate the effect of hybrid dye (ZHN-carmine) and cochineal carmine on mortadella color stability. The results were presented as average ± standard deviation, submitted to analysis of variance (one-way, ANOVA), and between treatments and storage time was used in the tables. Tukey's test was used when significant effects were found between treatments and/or during the storage, at significance level *p* < 0.05, using the Statistica 7.0 software.

## 3 Results and discussion

### 3.1 Hybrid dye (ZHN-carmine) characterization

**Fig. 1:**
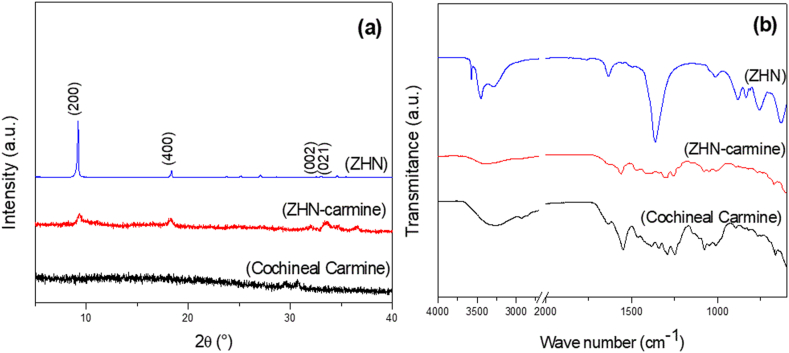
**a** X-ray diffractograms **b** FTIR spectra of solids

After the ion exchange reaction, the obtained solid (ZHN-carmine) showed a characteristic cochineal carmine color. The XDR pattern for ZHN (Fig. 1a) confirmed the Zn_5_(OH)_8_(NO_3_)_2_.2H_2_O structure, as per JPCDS File 24–1460. The basal distance value calculated using Bragg's Law was 9.65 Å for the hybrid dye (ZHN-carmine), in agreement with pure ZHN basal distance. The lamellar structure is proven by the presence of the peaks attributed to the reflection plane basal in the stacking direction of the layer (h00), as seen in Fig. 1a. The basal peaks observed in the 2*θ (degrees) region from 5 to 20°, showed a uniform distribution.

**Fig. 2:**
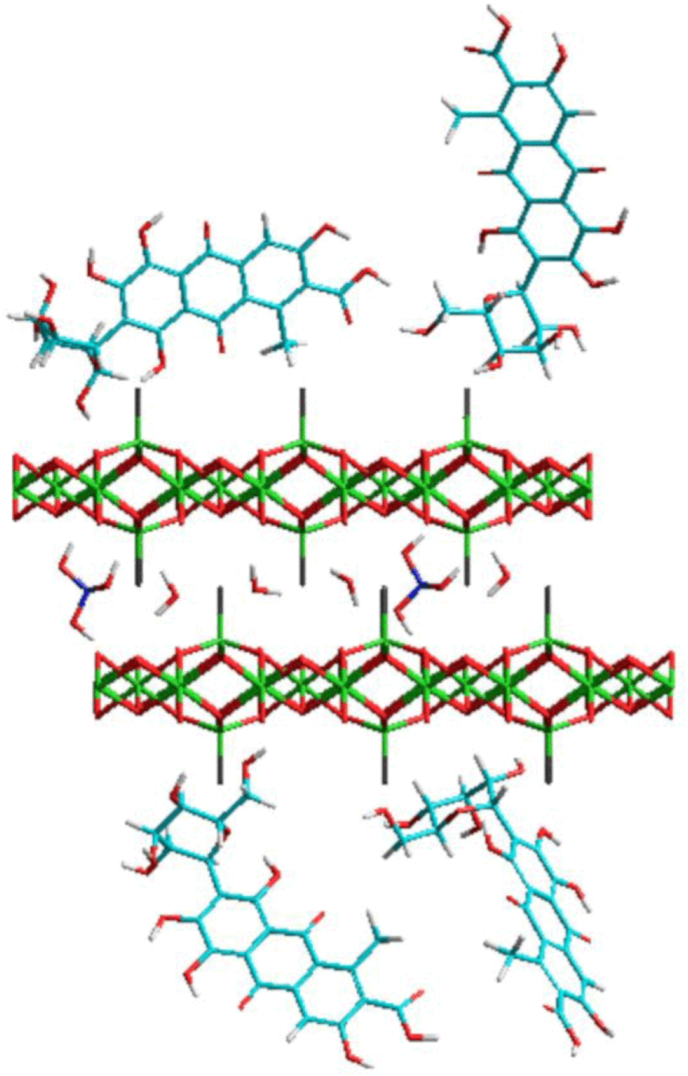
Scheme showing the adsorption of cochineal carmine in layered zinc hydroxide salt

It is important to point out that the hybrid dye (ZHN-carmine) XDR pattern (Fig. 1a) presents no pure cochineal carmine characteristic peaks, indicating the absence of pure starter reagent in the sample. Since no basal distance increase occurred, the ZHN-carmine diffraction pattern was similar to pure ZHN, although less crystalline, suggesting that the dye was adsorbed on the lamellar compound surface (Fig. 2) as also observed by Fournier et al. (2016) when adsorbing carmine dye in clay montmorillonite.

Cochineal carmine adsorption in ZHN was confirmed by vibrational spectroscopic. Peaks in the product spectrum (ZHN-carmine) attributed to the organic compound (cochineal carmine) and layered zinc hydroxide nitrate (ZHN) were noted. The ZHN FTIR spectra (Fig. 1b) showed typical nitrate vibrations band at 1363 cm^−1^ and H_2_O bending vibration at 1636 cm^−1^, as reported by Newman and Jones (1999). There is also broadband in the region of 3500 cm^−1^ assigned to the hydroxyl group vibrations that keeps multiple hydrogen bonds with water molecules, as well as a sharp band at 3575 cm^−1^ assigned to the hydroxyl groups in the layer, hence, with well-defined vibrational energy (Cursino et al., 2010; Newman and Jones 1999). The dye adsorption in the product (ZHN-carmine) (Fig. 1b) is confirmed by the presence of typical cochineal carmine bands as seen in Table 2.

**Table 2:** Main bands (FTIR) in the vibrational spectra of hybrid dye (ZHN-carmine) and cochineal carmine and respective assignments.

ZHN-carmine (cm^−1^)	Cochineal carmine (cm^−1^)	Assignment
1644	1643	*v* (C=O)
1564	1559	*v* (C=C)
1472	1469	*v* (C=C)
1311	1312	*v* (C=C) _ring_/δ (COH)
1254	1260	*v* (C–C)/δ (C–H)

Adapted (Bernardino, 2011, Whitney et al., 2006).

Furthermore, it was noted that the characteristic layered hydroxyls groups band at 3575 cm^−1^ did not appear in the ZHN-carmine product (Fig. 1b), indicating that the dye molecules interact with the layer surface hydroxyls, confirming the presence of cochineal carmine in the product. Furthermore, a significant nitrate ion bands reduction in the product (ZHN-carmine) (Fig. 1b) was verified, as previously observed in the ZHN azo dye adsorption study (Marangoni et al., 2009), suggesting that positive charges cause the dye molecules immobilization on the ZHN surface, probably replacing some nitrate anions on the outer surface and producing highly stable colored compounds.

**Fig. 3:**
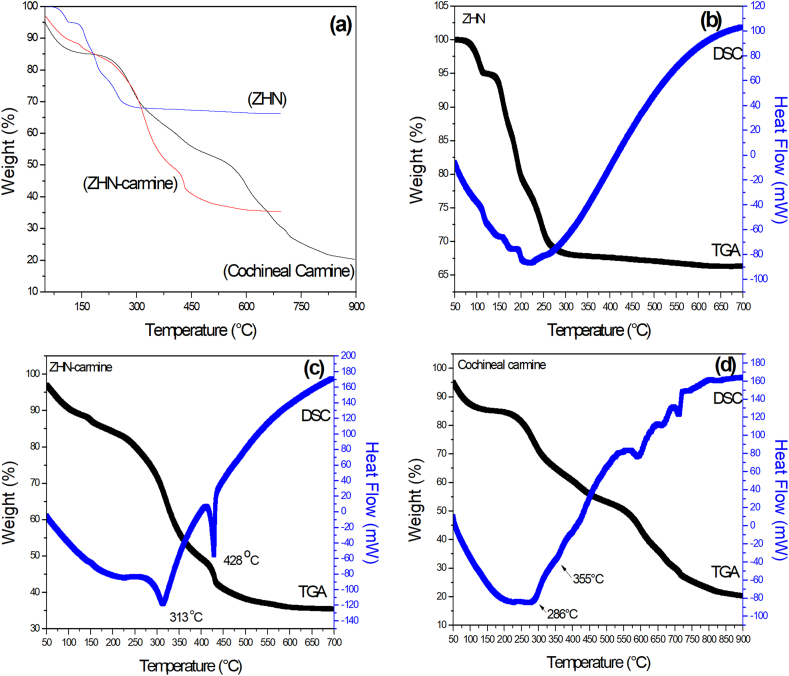
**a** Comparative of TGA curves for solids **b** Thermal analysis curves (TGA/DSC) of the ZHN **c** Hybrid dye (ZHN-carmine) and **d** Cochineal carmine

The TGA curves for layered zinc hydroxide salt (ZHN), hybrid dye (ZHN-carmine) and cochineal carmine are shown in Fig. 3a.

For all samples, a loss of mass linked to the removal of physiosorbed/intercalated water molecules ranging from 50 to 100 °C was observed (Fig. 3a). The thermal analysis curve for the ZHN compound (Fig. 3b) showed a loss of mass associated with the dehydroxylation process and the decomposition of the nitrate from the structure after 114 °C. The ZHN structure started its thermal degradation at 114 °C due to dehydroxylation forming Zn_3_(OH)_4_(NO_3_)_2_ and ZnO. Zn(NO_3_)_2_ and ZnO from Zn_3_(OH)_4_(NO_3_)_2_ dehydration were formed at 164 °C. Finally, Zn(NO_3_)_2_ was decomposed into ZnO, NO_2_, NO and O_2_ at 222 °C (Hongo et al., 2010).

For cochineal carmine, losses of mass linked to events at 288 °C and 355 °C were observed (Fig. 3d). The 288 °C might be linked to glucose pyrolysis with its maximum loss of mass at 355 °C, as reported by Norman et al. (2015). The other loss of mass noted may be due to thermal degradation of the carmine structure's aromatic part (Norman et al., 2015). A continuous loss of mass over 600 °C (Fig. 3d) and a final 20% residue was noted, which might be due to aluminum oxide formation from aluminum complexes (carmine lake). Such loss of mass over 600 °C was not seen in the TGA curve for the hybrid dye (ZHN-carmine) as it may have occurred due to pH changes during obtainment favoring the predominance of carminic acid rather than its aluminum complexes (Fournier et al., 2016). When adsorbing cochineal carmine in montmorillonite-type clay minerals observed a loss of mass linked to the organic matter oxidation between 150 °C and 500 °C, as observed for the (ZHN-carmine (Fig. 3c). For the hybrid dye (ZHN-carmine), such losses of mass linked to organic matter combustion are related to events at 313 °C and 428 °C. The hybrid material thermal stability increase compared to cochineal carmine occurred probably due to hydrogen bonds interaction formed between the hydroxyl groups from the layered zinc hydroxide salt and the dye (Norman et al., 2015).

The thermal behavior pattern change between cochineal carmine (Fig. 3d) and hybrid dye (Fig. 3c) suggests the occurrence of interactions between the layered zinc hydroxide salt and the dye. Moreover, a greater loss of mass observed for the ZHN-carmine hybrid dye (Fig. 3c), compared to ZHN (Fig. 3b), proved the cochineal carmine adsorption corroborating with the one observed by XRD and FTIR.

### 3.2 Mortadella color evaluation

L*, a*, and b* color parameters results for mortadella are seen in Table 3.

**Table 3:** Color parameters of the different mortadella formulations.

Parameters	Formulation	Storage period (days)					
		0	8	16	24	32	40
L*	M1	65.27 ± 0.70^aC^	68.69 ± 0.36^aA^	67.30 ± 1.05^aB^	65.74 ± 0.28^aC^	67.29 ± 0.40^aB^	64.94 ± 0.68^aC^
	M2	65.31 ± 0.62^aABC^	67.01 ± 0.84^bcA^	66.42 ± 0.42^aA^	63.70 ± 0.46^bCD^	65.86 ± 1.07^aAB^	62.27 ± 1.52^bD^
	M3	64.97 ± 0.72^aAB^	65.90 ± 1.11^cA^	64.25 ± 0.72^bAB^	63.67 ± 1.07^bBC^	64.33 ± 1.06^bAB^	62.27 ± 0.56^bC^
	M4	58.48 ± 0.89^bAB^	59.57 ± 0.24^dA^	58.91 ± 0.80^cAB^	58.18 ± 1.05^cAB^	58.13 ± 0.63^cB^	56.11 ± 0.35^cC^
a*	M1	9.69 ± 0.53^dA^	8.66 ± 0.33^dB^	8.48 ± 0.45^cB^	8.70 ± 0.08^dB^	8.52 ± 0.33^dB^	4.43 ± 0.33^dC^
	M2	15.03 ± 0.21^bA^	14.10 ± 0.22^bB^	13.26 ± 0.37^bCD^	13.76 ± 0.21^bBC^	12.57 ± 0.59^bD^	13.54 ± 0.32^bcBC^
	M3	11.26 ± 0.55^cB^	11.30 ± 0.53^cB^	12.78 ± 0.71^bA^	10.36 ± 0.31^cBC^	9.81 ± 0.41^cC^	12.59 ± 0.52^cA^
	M4	18.94 ± 0.43^aC^	20.30 ± 0.78^aB^	22.35 ± 0.48^aA^	20.36 ± 0.12^aB^	18.33 ± 0.26^aCD^	17.47 ± 0.47^aD^
b*	M1	12.79 ± 0.39^aD^	13.43 ± 0.18^aC^	13.55 ± 0.27^aBC^	14.08 ± 0.15^aB^	13.81 ± 0.22^aBC^	14.87 ± 0.39^aA^
	M2	11.99 ± 0.08^bAB^	12.49 ± 0.35^bA^	12.01 ± 0.17^bAB^	11.72 ± 0.18^cB^	11.98 ± 0.54^bAB^	12.00 ± 0.35^bAB^
	M3	12.40 ± 0.39^abBC^	13.48 ± 0.36^aA^	12.41 ± 0.16^bBC^	13.09 ± 0.37^bAB^	12.55 ± 0.47^bBC^	12.12 ± 0.43^bC^
	M4	9.32 ± 0.17^cB^	10.31 ± 0.15^cA^	10.32 ± 0.18^cA^	10.63 ± 0.34^dA^	9.58 ± 0.41^cB^	8.65 ± 0.22^cC^

M1. mortadella with curing salts and without dyes; M2. mortadella with curing salts and cochineal carmine; M3. mortadella without salts curing and with hybrid dye (ZHN-carmine); M4. mortadella without curing salts and with hybrid dye (ZHN-carmine) dissolved by ultrasound. Average ± Standard Deviation (n = 3) followed by different lowercase letters in the columns or different uppercase letters in the lines indicate a significant difference (*p* ≤ 0.05). Tukey's test.

All mortadella formulations showed differences in color during the storage period. No significant difference (*p* > 0.05) was observed for M1, M2 and M3 formulations L* values at zero time, but they differed (*p* < 0.05) from M4. The M1 sample with curing salts and without any dye presented a higher L* value (*p* < 0.05) - typical of a less intense coloring - at 8, 24, and 40 storage days, when compared to the other samples. However, M1 formulation did not show any significant difference (*p* > 0.05) from M2 and M3 at 0, 16 and 32 storage days. M4 formulation showed L* value significantly lower (*p* < 0.05) than the other samples, i.e., it has a more intense red color during 40 storage days. Moreover, a* values for the M4 formulation were significantly higher (*p* < 0.05) than those for the other samples during the experiment, reinforcing the evidence of a more visually intense red color characteristic. However, when comparing M1, M2 and M3 formulations, the a* value for M2 mortadella formulation was significantly higher (*p* < 0.05) than those for M1 and M3 formulations, for all storage times. It indicates a strong influence on color formation caused by curing salts addition, which acts synergistically with the cochineal carmine added. Furthermore, M1 and M2 formulations had a significant red color reduction (a*) during the storage period, as noted by the significant difference (p < 0.05) between 0 and 40 storage days period. Hybrid dye (ZHN-carmine) added to the M3 formulation stood out for its high stability when compared to other formulations. Overall, the b* value had less significant samples and storage times variations, showing the yellow color predominance in all formulations.

Baldin et al. (2016) reported the color of the control fresh sausage (with curing salt) and the fresh sausage with 4% cochineal carmine had a significant a* value reduction during 15 storage days, showing the red color instability caused by these color agents. It corroborated with the results found in this study for M1 and M2 formulations. On the other hand, the M3 formulation had its red color intensity increased with storage, and the M4 formulation had a red color intensity peak at 16 storage days. However, M4 formulation did not differ from 0 to 32 storage days, maintaining a more intense red color than the other formulations during the experiment. Such sharper color for M4 formulation may be related to the exfoliation effect caused by ultrasound when applied to the hybrid dye (ZHN-carmine). For layered zinc hydroxide salt, of which layers are stacked and connected by weak forces, the use of ultrasound may cause on individual sheets separation, consequently, causing the dye adsorption surface to be larger than the bulk three dimensional (3D) layered material (Marangon, 2008). Moreover, the ultrasound exfoliation provided a more homogeneous ZHN-carmine dispersion into the mortadella matrix. Such factors might have contributed to a greater red color intensity in mortadella.

It has been shown that polymeric films using layered compounds and azo dyes using ultrasound disaggregate and disperse the dye (Da Silva et al., 2013). Gonzalez et al. (2019) reported the use of natural hair dye enhanced with ultrasound and observed that ultrasound increased the natural dye's uptake and color intensity and decreased dyeing periods. Furthermore, Low et al. (2018), demonstrated that ultrasound may increase the dye adsorption's capacity and efficiency. Thus, the addition of hybrid dyes obtained from cochineal carmine adsorbed on layered zinc hydroxide salt (hybrid dye - ZHN-carmine) is a feasible alternative to red color stability in cooked mortadella.

## 4 Conclusion

The hybrid dye (ZHN-carmine) was obtained by cochineal carmine adsorption on layered zinc hydroxide salt and XRD suggest that the layered structure was consistent with pure ZHN. FTIR showed bands related with vibrational modes of cochineal carmine and the ZHN. TGA/DSC showed a change in the thermal behavior pattern between the cochineal carmine and the hybrid dye, demonstrating the occurrence of interactions between ZHN and dye. ZHN-carmine in mortadella showed a suitable red color stability in comparison with mortadella formulations added of curing salts and cochineal carmine. Furthermore, the ultrasonic treatment applied to HZN-carmine increased the red color intensity of the dye, allowing the use of lower percentages as commonly required on cooked meat product elaboration. This article showed the possibility of pink/reddish mortadella production without curing salts by using a hybrid dye ensuring the characteristic of cooked meat product.

## CRediT authorship contribution statement

**Gabriela Cavalca Ongaratto:** Methodology, Validation, Writing – original draft, Development of methodology, Conducting of experiments. Application of statistical, Validation of results/experiments. Preparation, creation and writing of the published article. **Gabriela Oro:** Methodology, Writing – original draft, Development or design of methodology. Conducting of experiments. Preparation, creation and writing of the published article. **Daneysa Lahis Kalschne:** Validation, Writing – original draft, Application of statistical. Validation of results/experiments. Preparation, creation and writing of the published article. **Ana Cristina Trindade Cursino:** Writing – original draft, Formulation of overarching research goals and aims. Oversight and leadership responsibility for the research activity planning and execution. Preparation, creation and writing of the published article. **Cristiane Canan:** Funding acquisition, Writing – original draft, Formulation of overarching research goals and aims. Acquisition of the financial support for the project leading to this publication. Oversight and leadership responsibility for the research activity planning and execution. Preparation, creation and writing of the published article.

## Declaration of competing interest

The authors declare that they have no known competing financial interests or personal relationships that could have appeared to influence the work reported in this paper.
